# A Computational Model of Limb Impedance Control Based on Principles of Internal Model Uncertainty

**DOI:** 10.1371/journal.pone.0013601

**Published:** 2010-10-26

**Authors:** Djordje Mitrovic, Stefan Klanke, Rieko Osu, Mitsuo Kawato, Sethu Vijayakumar

**Affiliations:** 1 IPAB, School of Informatics, University of Edinburgh, Edinburgh, United Kingdom; 2 ATR Computational Neuroscience Laboratories, Keihanna Science City, Kyoto, Japan; 3 National Institute of Information and Communications Technology, Kyoto, Japan; The University of Western Ontario, Canada

## Abstract

Efficient human motor control is characterized by an extensive use of joint impedance modulation, which is achieved by co-contracting antagonistic muscles in a way that is beneficial to the specific task. While there is much experimental evidence available that the nervous system employs such strategies, no generally-valid computational model of impedance control derived from first principles has been proposed so far. Here we develop a new impedance control model for antagonistic limb systems which is based on a minimization of uncertainties in the internal model predictions. In contrast to previously proposed models, our framework predicts a wide range of impedance control patterns, during stationary and adaptive tasks. This indicates that many well-known impedance control phenomena naturally emerge from the first principles of a stochastic optimization process that minimizes for internal model prediction uncertainties, along with energy and accuracy demands. The insights from this computational model could be used to interpret existing experimental impedance control data from the viewpoint of optimality or could even govern the design of future experiments based on principles of internal model uncertainty.

## Introduction

Suppose you are holding an umbrella in a stable upright position on a rainy day. This is an effortless task, however if suddenly a seemingly random wind gust perturbs the umbrella, you will typically stiffen up your arm trying to reduce the effects of the “unpredictable” perturbation. It is well established that the *central nervous system (CNS)* manages to change the mechanical properties (i.e., joint impedance) of limbs by co-activating antagonistic muscle pairs in response to specific task requirements. This is commonly referred to as *impedance control* [1], which has been explained as an effective strategy of the nervous system to cope with kinematic variability due to neuromuscular noise and environmental disturbances. Coming back to our umbrella example: If over time you realize the wind keeps blowing from the same direction, you expectedly will become more certain about the wind's destabilizing effect on your arm and you will gradually reduce the stiffness and you will possibly try to place the umbrella in a new stable position. This simple example shows intuitively how co-activation is linked to uncertainties that you may experience in your limb dynamics, and the main objective in this work is to develop a computational model that unifies the concepts of *learning*, *uncertainty* and *optimality* in order to understand impedance control in a principled fashion.

A large body of experimental work has investigated the motor learning processes in tasks under changing dynamics conditions [2], [3], [4], revealing that subjects generously make use of impedance control to counteract destabilizing external force fields (FF). Indeed impedance modulation appears to be, to some extent, governed by preservation of metabolic cost [2] in that subjects do not just naively stiffen up their limbs but rather learn the optimal mechanical impedance by predictively controlling the magnitude, shape, and orientation of the endpoint stiffness in the direction of the instability. In the early stage of dynamics learning, humans tend to increase co-contraction and as learning progresses in consecutive reaching trials, a reduction in co-contraction along with a simultaneous reduction of the reaching errors made can be observed [4]. These learning effects are stronger in stable FF (i.e., velocity-dependent) compared to unstable FF (i.e., divergent), which suggests that impedance control is connected to the learning process with internal dynamics models and that the CNS employs co-activation to increase task accuracy in early stages of learning, when the internal model is not adequately accurate yet [5], [6].

Notably limb impedance is not only controlled during adaptation but also in tasks under stationary dynamics conditions. Studies in single and multi-joint limb reaching movements revealed that stiffness is increased with faster movements [7], [8] as well as with higher positional accuracy demands [9], [10]. Under such conditions, higher impedance is linked to reducing the detrimental effects of neuromotor noise [11], which exhibits large control signal dependencies [12]. Similar to our umbrella example, in the stationary case, the impedance can be linked to uncertainty which here however arises from internal sources.

Many proposed computational models have focused on the biomechanical aspects of impedance control [13], [14] or have provided ways to reproduce accurately observed co-activation patterns for specific experiments [4], [15]. While such models are important for the phenomenological understanding of impedance control, they do not provide principled insights about the origins of a wider range of phenomena, i.e., they cannot predict impedance control during both, stationary *and* adaptation experiments. Furthermore, it is not clear how impedance control can be formalized within the framework of optimal control, which has been immensely successful in the study of neural motor control. More specifically impedance control (i.e., muscle co-contraction) and energy preservation seem to be opposing properties and it has not been shown yet from a computational perspective how these properties can be unified in a single framework.

Here we develop a new computational theory for impedance control which explains muscle co-activation in human arm reaching tasks as an emergent mechanism from the first principles of optimality. Our model is formalized within the powerful theory of *stochastic Optimal Feedback Control (OFC)* [16]. However unlike previous OFC formulations that require a closed analytical form of the plant dynamics model, we postulate that this internal dynamics model is acquired as a motor learning process based on continuous sensorimotor feedback. From a computational perspective, this approach offers *three significant improvements* over state-of-the art OFC models for neuromotor control:

1. We can model *adaptation processes* due to modified dynamics conditions from an optimality viewpoint, without making prior assumptions about the source or nature of the novel dynamics.
2. Dynamics learning further provides us with means to model *prediction uncertainty* based on experienced stochastic movement data; we provide evidence that, in conjunction with an appropriate antagonistic arm and realistic *motor variability* model, impedance control emerges from a stochastic optimization process that minimizes these prediction uncertainties of the learned internal model.
3. By formalizing impedance control within the theory of stochastic OFC, we overcome the fundamental inability of energy based optimization methods to model co-contraction. Notably, in our model, co-contraction is achieved without changing the standard energy based cost function since the uncertainty information is contained in the learned internal dynamics function as a stochastic term. Therefore, the trade-off between energy preservation and co-contraction is primarily governed by the learned uncertainty of the limb system and by the accuracy demands of the task at hand.

We verify our model by comparing its predictions with two classes of published impedance control experiments: Firstly, stationary reaching experiments where accuracy or velocity constraints are modulated and secondly, tasks involving adaptation towards external FF. The results from single-joint elbow motion show, as predicted by the theory, that we can replicate many well-known impedance control phenomena from the first principles of optimality and *conceptually* explain the origins of co-contraction in volitional human reaching tasks.

## Results

Stochastic OFC has been shown to be a powerful theory for interpreting biological motor control [16]–[19], since it unifies motor costs, expected rewards, internal models, noise and sensory feedback into a coherent mathematical framework [20]. For the study of impedance control, optimality principles are well motivated given the fact that humans show energy and task optimal impedance modulation [2]. Formulating a reaching task in this framework requires a definition of a performance index (i.e., cost-function) to minimize for, typically including reaching error, end-point stability and energy expenditure. Other proposed cost functions often describe kinematic parameters only [21] or dynamics parameters based on joint torques [22], both of which do not allow a study of joint impedance at the level of muscle activations.

In addition to the cost function, an internal model needs to be identified, which represents the (possibly stochastic) dynamics function of the controlled arm (*see* *Methods*). Indeed, internal models play a key role in efficient human motor control [23] and it has been suggested that the motor system forms an internal forward dynamics model to compensate for delays, uncertainty of sensory feedback, and environmental changes in a predictive fashion [24], [25]. Following this motivation, we build our internal dynamics model based on a motor learning process from continuous sensorimotor plant feedback. Such a learned internal model offers two advantages: First, it allows for model adaptation processes by updating the internal model with newly available training data from the limbs [26]. Second, this training data contains valuable stochastic information about the dynamics and uncertainties therein. As motivated in the introduction, the uncertainty could originate from both internal sources (e.g., motor noise) and from environmental changes during adaptation tasks. The crucial point here is that learning a stochastic internal model enables a *unified treatment* of all the different types of perturbations, the effects of which are visible as predictive uncertainties.

**Figure 1:**
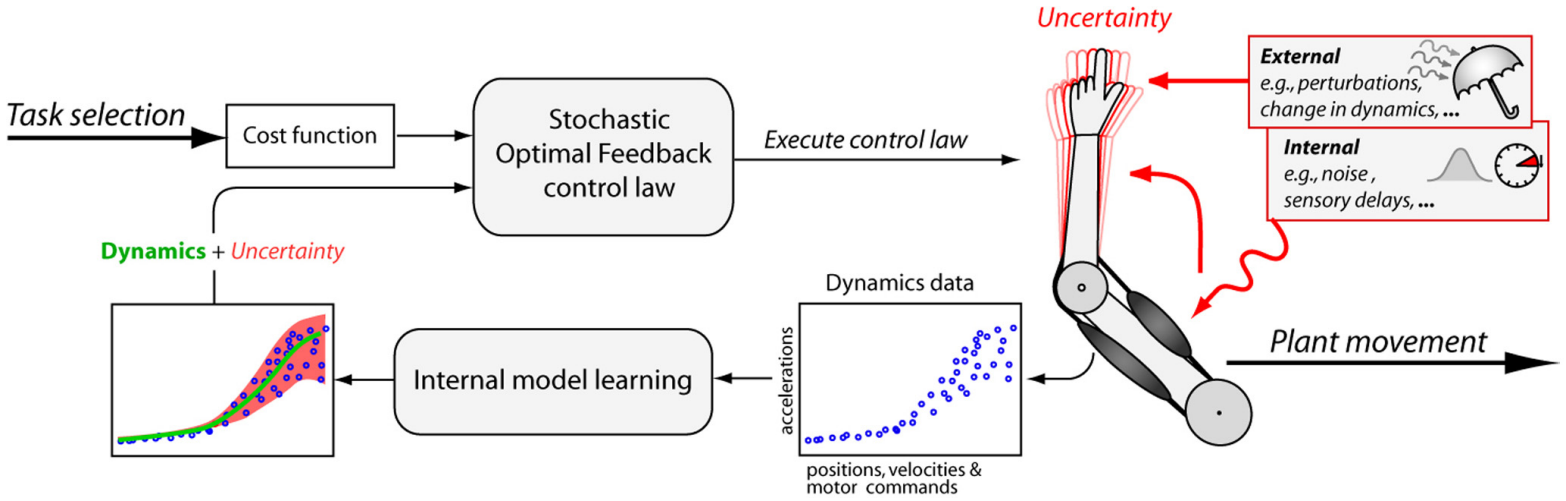
Schematic representation of our OFC-LD approach. The optimal controller requires a cost function, which here encodes for reaching time, endpoint accuracy, endpoint velocity (i.e., stability), and energy efficiency. Further a forward dynamics function is required, which in OFC-LD is *learned* from plant feedback directly. This learned internal dynamics function not only allows us to model changes in the plant dynamics (i.e., adaptation) but also encodes for the uncertainty in the dynamics data. The uncertainty itself, visible as kinematic variability in the plant, can originate from different sources, which we here classify into *external* sources and *internal* sources of uncertainty. Most notably OFC-LD identifies the uncertainty directly from the *dynamics data* not making prior assumptions about its source or shape.

By incorporating this model into the optimal control framework (**Fig. 1**), we can formulate *OFC with learned dynamics (OFC-LD)* which, besides minimizing energy consumption and end point error, incorporates the prediction uncertainties into the optimization process [27]. Such an assumption is appropriate since humans have the ability to learn not only the dynamics but also the stochastic characteristics of tasks, in order to optimally learn the control of a complex task [28], [29]. Algorithmically OFC-LD relies on a supervised learning method that has the capability to learn heteroscedastic (i.e., localized) variances within the state-action space of the arm (*see* *Methods*).

### Modelling plausible kinematic variability

The human sensorimotor system exhibits highly stochastic characteristics due to various cellular and behavioral sources of variability [30] and a complete motor control theory must contend with the detrimental effects of signal dependent noise (SDN) on task performance. Generally speaking SDN in the motor system leads to kinematic variability in the arm motion and in attempts to incorporate this stochastic information into the optimization process, earlier models assumed a noise process, what we here refer to as *standard SDN*, that monotonically increased with the control signal [12], [31]. Those models have been successful in reproducing important psychophysical findings [32], [33]; however, in essence they simply scale the resulting *kinematic variability (KV)* with the control signal's magnitude and ignore the underlying noise-impedance characteristics of the musculoskeletal system [34], [35]. Consequently such methods, like all energy based methods, are only concerned with finding the lowest muscle activation possible, penalizing large activations and disallowing co-contraction. Generally, we define co-contraction as the minimum of two antagonistic muscle signals 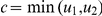 [5]. However, experimental evidence suggests that the CNS “sacrifices” energetic costs by co-contracting under certain conditions to increase impedance. But how can we model plausible kinematic variability arising from SDN?

Kinematic variability in human motion originates from a number of inevitable sources of internal force fluctuations [11], [30]. SDN [31] as well as joint impedance [36] increase monotonically with the level of muscle co-activation leading to the paradoxical situation that muscles are the source of force fluctuation and at the same time the means to suppress its effect by increasing joint impedance [34], [35]: Since SDN propagates through the muscle dynamics and impedance of the arm leading to kinematic variability, impedance can be changed to modulate the kinematic effects of the motor noise. Consequently, even though higher impedance implies higher co-activation and thus larger SDN levels in the muscles, in humans it leads to smaller kinematic variability [34].

**Figure 2:**
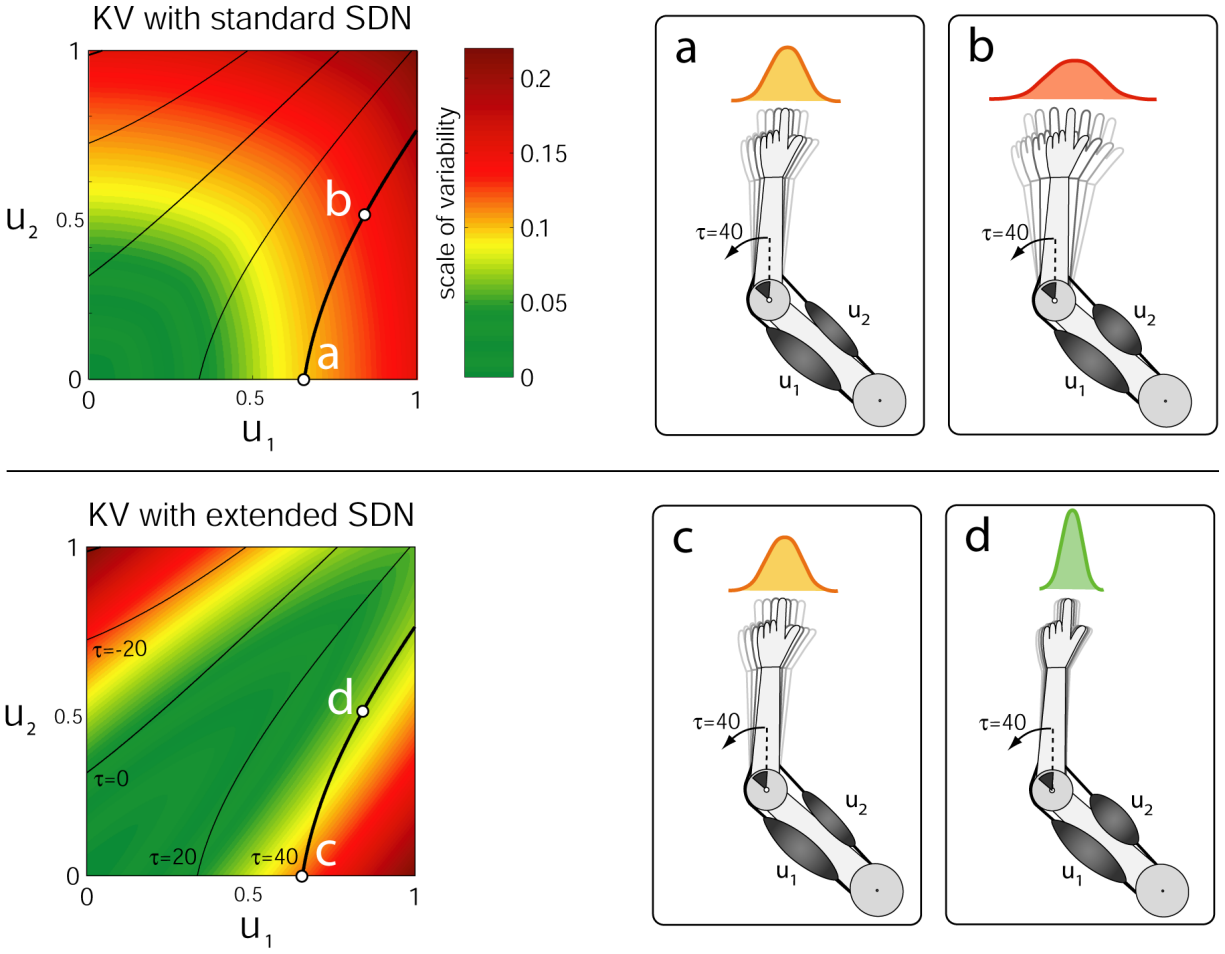
Illustration of the effects of standard and extended SDN on kinematic variability in the end-effector. Standard SDN scales proportionally to the muscle activation, whereas the extended SDN takes into account the stabilizing effects of higher joint impedance when co-contracting (see Methods), producing a “valley of reduced SDN” along the co-contraction line 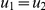. The colors represent the noise variance as a function of muscle activations, whereas the dark lines represent muscle activations that exert the same joint torque computed for joint angle position 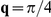. (a) Only muscle 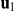 is activated, producing 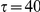 Nm joint torque with a Gaussian kinematic variability of 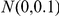. (b) The same torque with higher co-contraction produces significantly higher kinematic variability of 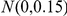 under standard SDN. (c) Same conditions as in (a) in the case where only muscle 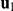 is activated. In contrast to (b) the extended SDN in (d) favors co-contraction leading to smaller kinematic variability of 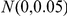 and to more stable reaching.

In order to account for this important property of human limbs, detailed muscular simulation models [35] have been proposed that showed that muscle-co-contraction has a similar effect to a low-pass filter to the kinematic variability. This is achieved by a relatively complex motor unit pool model of parallel Hill-type motor units that model realistic motor variability. In this work, since we are primarily interested in the computational aspects of impedance control, we increase the realism of our arm model by imposing an appropriate model of kinematic variability based on physiological observations, i.e., that the kinematic variability is reduced for more highly co-contracted activation patterns (*see* *Methods* *and* *Fig. 2*). Please note that this extended SDN models the kinematic variability that would results from a realistic antagonistic limb system (that suffers from SDN) and acts as an appropriate surrogate to employing a very detailed biophysical model. Indeed, the assumptions made in the extended SDN are supported by numerous experimental and computational results [34], [35] and furthermore, provide the computational ingredients that enable stochastic OFC framework to overcome the “inability” to co-activate anatagonistic muscle pairs. Most importantly, for the presented optimization and learning framework per se, it is irrelevant how the kinematic variability is modeled within the simulation (i.e, extended SDN versus highly detailed simulation model) since the learner acquires the stochastic information from plant data directly. For illustrative purposes, we present the differences between kinematic variability that arise from standard SDN (**Fig. 2a, b**) and from extended SDN (**Fig. 2c, d**) as produced by a single joint two-muscle model of the human elbow.

### Uncertainty driven impedance control

In the case when the internal model is learned from a plant with stochastic characteristics similar to the extended SDN model, the *prediction uncertainty* reflects the limb's underlying noise-impedance characteristics, i.e., the fact that co-contraction reduces variability. The optimal control policy therefore should favor co-contraction in order to reduce the negative effects of the SDN.

**Figure 3:**
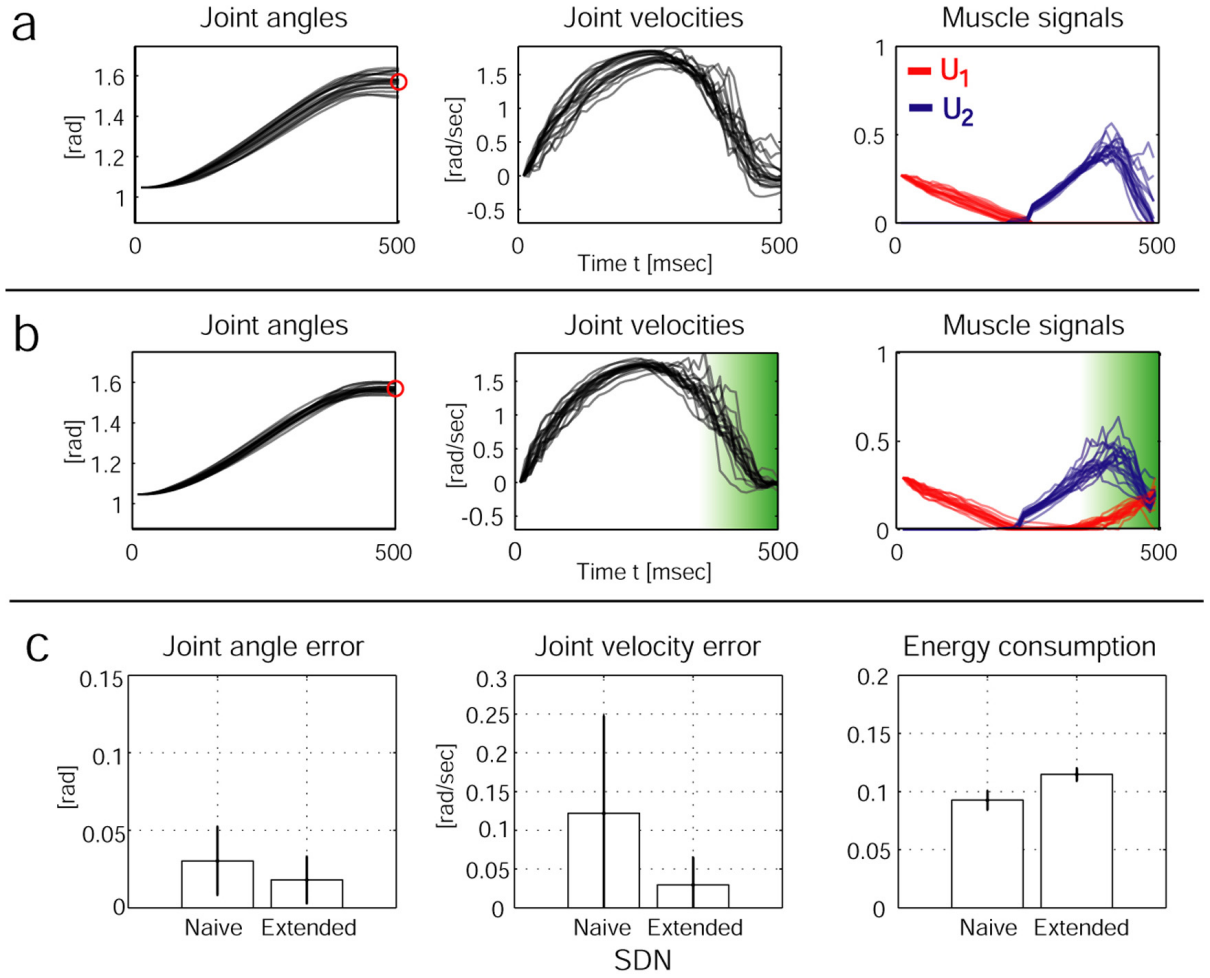
Comparison of the results from stochastic OFC using standard SDN (a) and extended SDN (b). We performed 50 OFC reaching movements (only 20 trajectories plotted) under both stochastic conditions. The shaded green area indicates the region and amount of co-contraction in the extended SDN solution. The plots in (c) quantify the results (mean +/− standard deviation). Left: average joint angle error (absolute values) at final time T = 500 msec. Middle: Joint angle velocity (absolute values) at time T. Right: integrated muscle commands (of both muscles) over trials. The extended SDN outperforms the reaching performance of the standard SDN case at the expense of higher energy consumption.

In order to test this hypothesis, we compared two stochastic OFC-LD solutions using internal dynamics models learned from a plant that either exhibits standard (**Fig. 3a**) or extended SDN (**Fig. 3b**). The optimal strategy found in this case is to try to avoid large commands **u** mostly at the end of the movement, where disturbances can not be corrected anymore. Notably, as is evident from **Fig. 3a** (right), there is still no co-contraction at all. In the extended noise scenario, a solution is found that minimizes the negative effects of the noise by increasing co-contraction at the end of the motion (see **Fig. 3b** (right)). The results reveal that the extended SDN performs significantly better than the standard SDN in terms of end point accuracy and end point velocity (**Fig. 3c**). By minimizing the uncertainty in a scenario with a neurophysiologically realistic model of kinematic variability, impedance control naturally emerges from the optimization, producing the characteristic tri-phasic control signals observed in human reaching [37]. Next we present the model's prediction on a set of well known impedance control phenomena in human arm reaching under stationary dynamics conditions.

### Impedance control for higher accuracy demands

**Figure 4:**
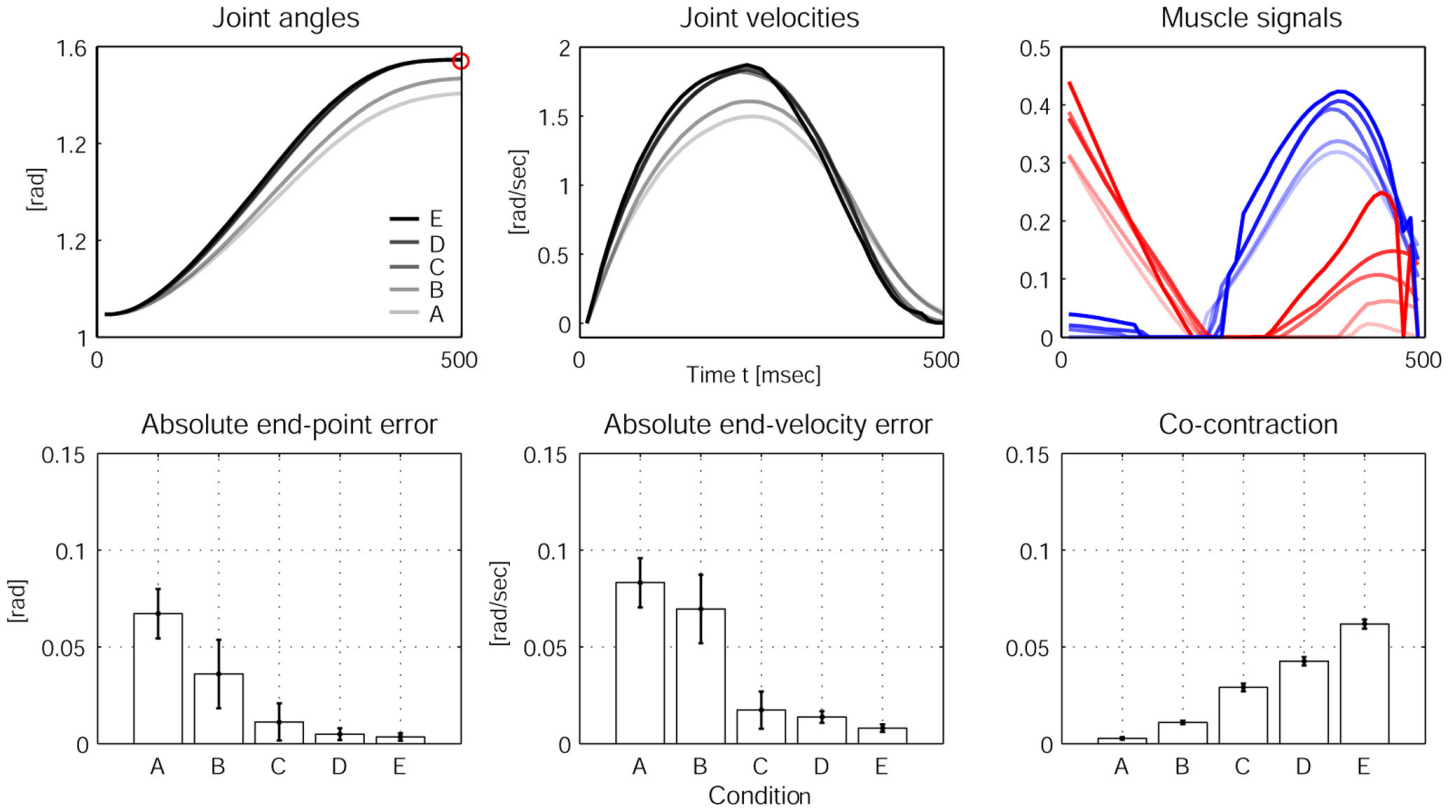
Experimental results from stochastic OFC-LD for different accuracy demands. The first row of plots shows the averaged joint angles (left), the averaged joint velocities (middle) and the averaged muscle signals (right) over 20 trials for the five conditions A, B, C, D, and E. The darkness of the lines indicates the level of accuracy; the brightest line indicates condition A, the darkest condition E. The bar plots in the second row average the reaching performance over 20 trials for each condition. Left: The absolute end-point error and the end-point variability in the trajectories decreases as accuracy demands are increased; Middle: End-point stability also increases (demonstrated by decreasing error in final velocities); Right: The averaged co-contraction integrated during 500 msec increases with higher accuracy demands, leading to the reciprocal relationship between accuracy and impedance control as observed in humans.

Although energetically expensive, co-contraction is used by the motor system to facilitate arm movement accuracy in single-joint [34] and multi joint reaching [9]. Experimentally, an inverse relationship between target size and co-contraction has been reported. As target size is reduced, co-contraction and joint impedance increases and trajectory variability decreases. As in the CNS, our model predicts the energetically more expensive strategy to facilitate arm movement accuracy. **Fig. 4** shows the predictions of our model for five conditions ranging from low accuracy demands (A) to high accuracy demands (E) (*see* *Methods*). In condition (A), very low muscle signals suffice to satisfy the low accuracy demands, while in the condition (E), much higher muscle signals are required, which consequently leads to higher co-contraction levels. A similar trend of increased muscle activation has been reported experimentally [38]. From an optimal control perspective, an increase in accuracy demands means also that influence of the stochasticity in the dynamics is weighted higher, which leads to a reduction of the relative importance of the energy efficiency in the cost function.

### Impedance control with increased velocities

**Figure 5:**
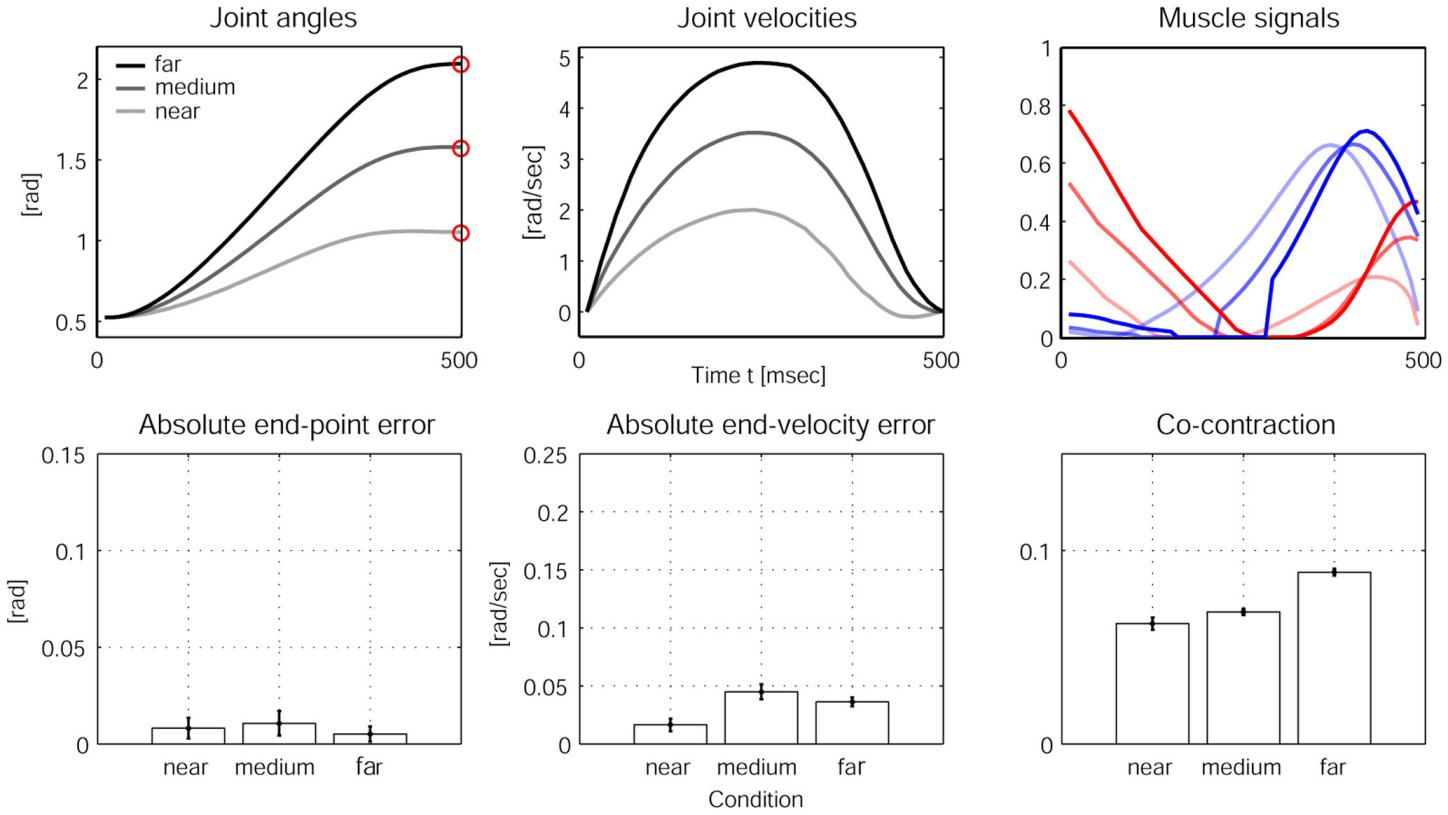
Experimental results from stochastic OFC-LD for different peak joint velocities. The first row of plots shows the averaged joint angles (left), the averaged joint velocities (middle) and the averaged muscle signals (right) over 20 trials for reaches towards the three target conditions “near”, “medium” and “far”. The darkest line indicates “far”, the brightest indicates the “near” condition. The bar plots in the second row quantify the reaching performance averaged over 20 trials for each condition. The end-point errors (left) and end-velocity errors (middle) show good performance but no significant differences between the conditions, while co-contraction during the motion as expected increases with higher velocities, due to the higher levels of muscle signals.

Next we test our model predictions in conditions where the arm peak velocities are modulated. Humans increase co-activation as well as reciprocal muscle activation with maximum joint velocity and it was hypothesized that the nervous system uses a simple strategy to adjust co-contraction and limb impedance in association with movement speed [8], [39]. The causalities here are that faster motion requires higher muscle activity which in turn introduces more noise into the system, the negative effects of which can be limited with higher joint impedance. Assuming that the reaching time and accuracy demand remains constant, peak velocities can be modulated using targets with different reaching distance (*see* *Methods*). The results in **Fig. 5** show that the co-contraction increases for targets that are further away and have a higher peak velocity. The reaching performance remains good for all targets, while there are minimal differences in end-point and end-velocity errors between conditions.

The presented stationary experiments exemplified how the proposed stochastic OFC-LD model can explain the emergence of impedance control from a computational perspective. In both experiments, OFC-LD increasingly makes use of co-contraction in order to fulfill the changing task requirements by choosing “more certain” areas of the internal dynamics model. While in the first case, this is directly caused by the higher accuracy demand, in the second case, the necessarily larger torques would yield less accuracy without co-contraction. Typically, “M-shaped” co-contraction patterns are produced, which in our results were biased towards the end of the motion. The bias can be attributed to the nature of the finite-horizon optimal control solution, which penalizes the effects of noise more towards the end of the motion, i.e., near the target state. Notably, M-shaped co-activation patterns have been reported experimentally [40] linking the magnitude of co-activation directly to the level of reciprocal muscle activation.

### Impedance control during adaptation

Adaptation paradigms, typically using a robotic manipulandum, have been a very fruitful line of experimental research [41]. In such setups, subjects are first thoroughly trained under normal reaching conditions (null field (NF)) and then, their adaptation process to changed dynamics (e.g., novel FF) is studied in consecutive reaching trials. While we have already linked uncertainties from internal sources to impedance modulation, the force field paradigm introduces additional “external” uncertainties of often larger magnitude. As we show next, in the spirit of the umbrella example from the introduction, the notion of internal model uncertainties becomes important for impedance control during adaptation.

A particular benefit of our model is that it employs an entirely data driven (learned) internal dynamics and noise model, meaning it can model changes in the environmental conditions. In the FF catch trial (the first reach in the new FF condition), the arm gets strongly deflected, missing the target because the internal model 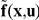 cannot yet account for the “spurious” forces of the FF. However, using the resultant deflected trajectory as training data and updating the dynamics model online brings the arm nearer to the target with each new trial as the internal model predictions become more accurate for the new condition.

**Figure 6:**
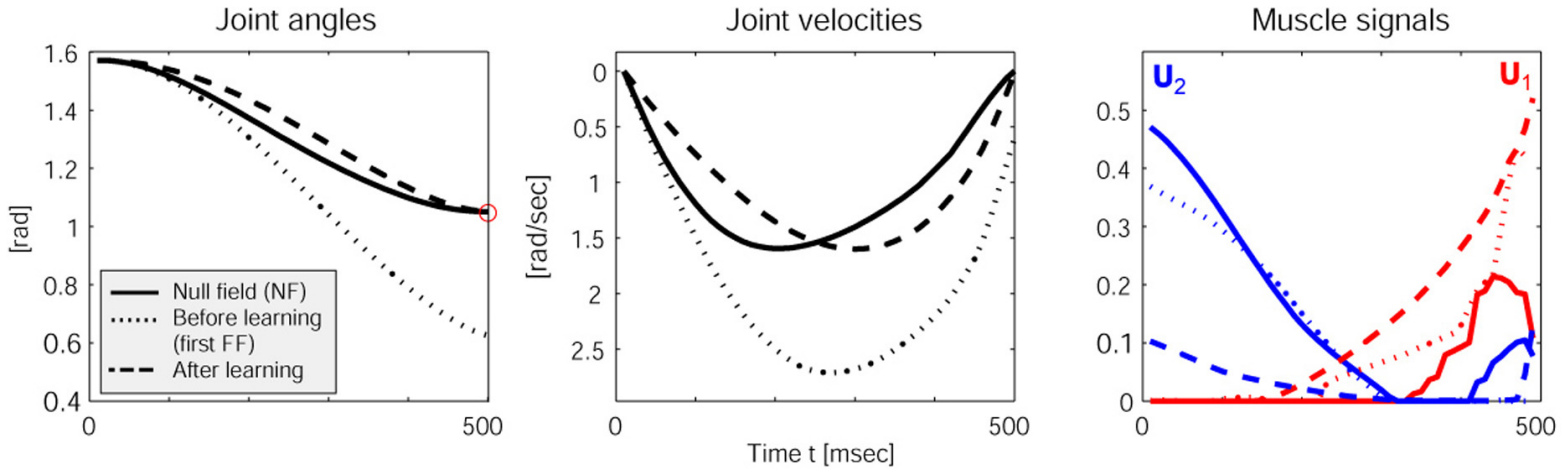
Optimal reaching movement, before, during and after adaptation. Clearly the solution is being re-optimized with the learned dynamics (including the FF).

Our adaptation experiment starts with 5 trials in a NF condition, followed by 20 reaching trials in the FF condition (*see* *Methods*). For each trial, we monitored the muscle activations, the co-contraction and the accuracy in the positions and velocities. Since the simulated system is stochastic and suffers from extended SDN, we repeated the adaptation experiment 20 times under the same conditions and averaged all results. **Fig. 6** aggregates these results. We see in the kinematic domain (left and middle plots) that the adapted optimal solution differs from the NF condition, suggesting that a re-optimization takes place. After the force field has been learned, the activations for the extensor muscle 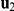 are lower and those for the flexor muscle 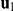 are higher, meaning that the optimal controller makes use of the supportive force field in positive x-direction. Indeed these results are in line with recent findings in human motor learning, where Izawa and colleagues [42] presented results that suggest that such motor adaptation is not just a process of perturbation cancellation but rather a re-optimization w.r.t. motor cost and the novel dynamics.

**Figure 7:**
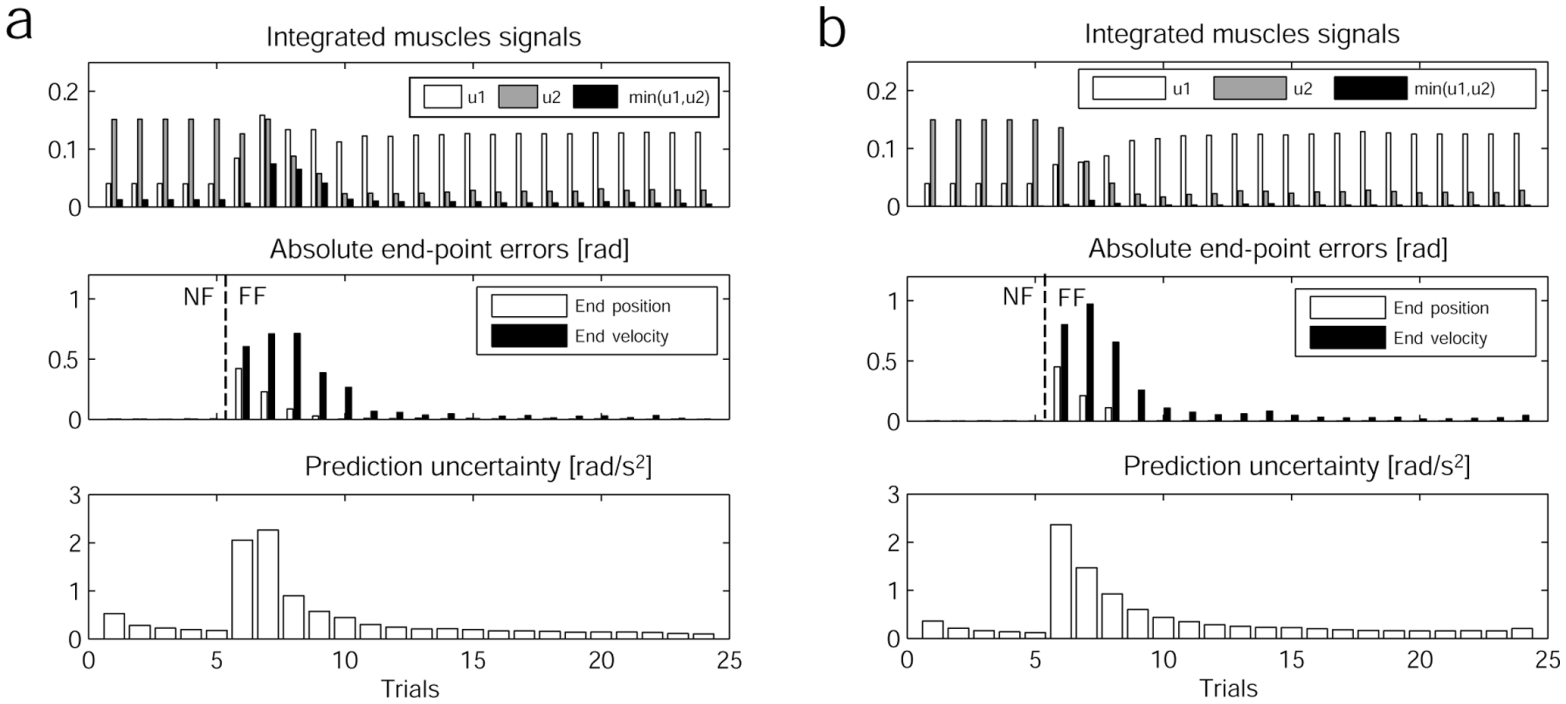
Adaptation results. (a) Accumulated statistics during 25 adaptation trials using stochastic OFC-LD. Trials 1 to 5 are performed in the NF condition. Top: Muscle activations and co-contraction integrated during 500ms reaches. Middle: Absolute joint errors and velocity errors at final time *T* = 500*ms*. Bottom: Integrated (internal model) prediction uncertainties along the current optimal trajectory, after this has been updated. (b) The same statistics for the adaptation using deterministic OFC-LD, meaning no uncertainty information is used for the optimization. This leads to no co-contraction and therefore worse reaching performance during adaptation.

To analyze the adaptation process in more detail, **Fig. 7a** presents the integrated muscle signals and co-contraction, the resultant absolute end-point and end-velocity errors and the prediction uncertainty of the internal model (i.e., heteroscedastic variances) during each of the performed 25 reaching trials. The prediction uncertainty was computed after each trial with the updated dynamics along the current trajectory. The first five trials in the NF condition show approximately constant muscle parameters along with good reaching performance and generally low prediction uncertainties. Even in the NF condition, the learning further reduces the already low uncertainty. In trial 6, the FF catch trial, the reaching performance drops drastically due to the novel dynamics. This also increases the prediction uncertainty since the input distribution along the current trajectory has changed and “blown up” the uncertainty in that region. Consequently, the OFC-LD algorithm now has to cope with increased uncertainty along that new trajectory. These can be reduced by increasing co-contraction and therefore, entering lower noise regions, which allow the algorithm to keep the uncertainty lower and still produce enough joint torque. For the next four trials, i.e. trials 7 to 10, the co-activation level stays elevated while the internal model gets updated, which is indicated by the change in reciprocal activations and improved performance between those trials. After the 11th trial, the co-contraction has reduced to roughly the normal NF level and the prediction uncertainty along the trajectory is fairly low (<1) and keeps decreasing, which highlights the expected connection between impedance and prediction uncertainty. A further indication for the viability of our impedance control model is supported with a direct comparison to the deterministic case. We repeated the same adaptation experiment using a deterministic OFC-LD implementation, meaning the algorithm ignored the stochastic uncertainty information available for the optimization (**Fig. 7b**). For the deterministic case, one can observe that virtually no co-contraction during adaptation is produced. This leads generally to larger errors in the early learning phase (trial 6 to 10), especially in the joint velocities. In contrast, for the stochastic algorithm, the increased joint impedance stabilizes the arm better towards the effects of the FF and therefore, produces smaller errors.

The comparison of the stochastic versus deterministic adaptation example highlights the necessity and importance of the optimal controller's ability to learn the stochastic information structure of the motor system in the NF condition from observations, i.e., the structure of the kinematic variability resulting from the extended SDN, such that it can be used to achieve more stable reaching performance during adaptation tasks.

## Discussion

We present a computational model for joint impedance control that is stable towards internal and external fluctuations. Our model is based on the fundamental assumption that the CNS, besides optimizing for energy and accuracy, minimizes the expected uncertainty from its internal dynamics model predictions. Indeed this hypothesis is supported by numerous experimental findings in which the CNS sacrifices energetic costs of muscles to reach stability through higher joint impedance in uncertain conditions. We showed that, in conjunction with an appropriate antagonistic arm and SDN model, the impedance control strategy emerges from first principles as a result of an optimization process that minimizes for energy consumption and reaching error. Unlike previous OFC models, here, the actor utilizes a learned dynamics model from data that are produced by the limb system directly. The learner incorporates the contained kinematic variability, here also termed noise, as prediction uncertainty which is represented algorithmically in form of heteroscedastic (i.e., localized) variances. With these ingredients, we formulated a stochastic OFC algorithm, called OFC-LD that uses the learned dynamics and the contained uncertainty information. This generic model for impedance control of antagonistic limb systems is solely based on the quality of the learned internal model and therefore, leads to the intuitive requirement that impedance will be increased in cases where the actor is uncertain about the model predictions. The simulated model predictions agree with several well-known experimental findings from human impedance control and, for the first time, does so from first principles of optimal control theory.

Even though the proposed framework here makes use of specific computational techniques for nonlinear OFC (i.e., ILQG) and heteroscedastic learning (i.e., LWPR), alternative planning and learning methods could be applied. The key novelty of our computational model is that it unifies the concepts of *energy-optimality*, *internal model learning* and *uncertainty* to a principled model limb impedance control.

In our model, we create a unified treatment of the various sources of kinematic variability (sensorimotor noise, external perturbations, systematic load or force fields etc.) by incorporating this into a *perceived error* in internal model predictions. Indeed, many human motor behaviors can be explained by stochastic optimal control models that minimize the impact of motor noise [12], [43], [44]. While exploiting this in our framework, the structured stochasticity provides additional information about the system dynamics and the emergent impedance control may be a further indication of the possible constructive role of noise in the neuromotor system [30]. The methodology we suggest for optimal exploitation of sensorimotor stochasticity through learning is a generic principle that goes beyond the modeling of signal dependent sources of noise but can be generalised to deal with other kinds of control or state dependent uncertainties. An example would be uncertainties that depend on the arm position or current muscle lengths.

In the presented optimal control formulation, the uncertainty of the internal model predictions are included in the dynamics formulation as a stochastic term. Alternatively, one could introduce uncertainty as an additional “uncertainty term” into the cost function. The advantage of our approach is that uncertainty or kinematic variability is modeled at its origin, i.e., in the dynamics of the system. Therefore, we can not only retain the original cost function description but also take into account the time course of the movement and therefore, minimize directly for the “detrimental effects” of the uncertainty specifically to our planning time horizon as shown in the stationary experiments.

While we have suggested a computational framework to bridge the gap between optimal control and co-activation, there is still limited knowledge about the neural substrate behind the observed optimality principles in motor control [20]. Our model is a first attempt to formalize the origins of impedance control in the CNS from first principles and many modifications could be considered. For instance, so far only process noise is modeled and observation noise is ignored entirely. This is a simplification of real biological systems, in which large noise in the observations is present, both from vision and proprioceptive sensors. Computationally, there are methods for solving nonlinear stochastic OFC with partial observability [27], [33], which could be employed for such a scenario. Experimentally, however, no clear connection between observation noise and impedance control has been established.

While this work has focused on the origins of impedance phenomena rather than on a faithful reproduction of published patterns, the predictions of the adaptation experiments are in remarkable agreement with previous findings [41], [42]. Furthermore, to the best of our knowledge, this is the first computational model to predict impedance control for both, stationary and adaptation experiments. Most importantly, our model is able to qualitatively predict the time course of impedance modulation across trials depending on the “learnability” of the external perturbations.

There are several further issues that warrant careful consideration. First, we make the fundamental assumption that the impedance control is achieved in a predictive fashion, i.e., through feedforward commands only, while there is experimental evidence that task specific reflex modulation also increases limb impedance in task relevant directions [45]. A viable route for future studies in this direction is to investigate parallels of the feedback gain matrix **L** and reflex modulation observed in humans. Second, impedance control in humans is not only achieved through voluntary muscle co-contraction but also through the anatomical routing of tendons and muscles [46]. Such increase in limb impedance is not governed by neural commands directly but rather emerges through the inherent biomechanical limb properties. Such effects could be incorporated into OFC-LD for example by using models of human limbs that exhibit more realistic biomechanical properties. The effects of this would be visible in the training data i.e., a smaller kinematic variability in certain regions of the state space and therefore narrower confidence bounds in LWPR.

The results presented in this paper can be expected to scale to higher dimensional systems, since impedance control seems to originate from the antagonistic muscle structure in the joint-space domain [15], [39], [47]. It is well known that humans also employ mechanisms other than co-contraction to increase task-specific limb stability. For example, human subjects extensively use kinematic limb relocation strategies to improve end-effector stiffness for specific tasks [48]. It remains to be seen whether the minimum uncertainty approach has the capability to explain these and other important multi-joint impedance phenomena such as the end-effector stiffness that is selectively tuned towards the directions of instability [2], [10]. Nevertheless our general model of impedance control may serve as an important step towards the understanding of how the CNS modulates impedance through muscle co-contraction.

## Methods

### An antagonistic arm model for impedance control

The nonlinear dynamics of our human elbow is based on standard equations of motion. The joint torques 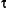 are given by
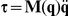
with joint angles 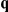, accelerations 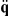, inertia matrix **M**. The joint torque produced by the antagonistic muscle pair is a function of its muscle tension **t** and of the moment arm **A**, which for simplicity's sake is assumed constant. The effective joint torque from the muscle commands 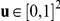 is given by
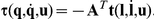
The muscle lengths **l** depend on the joint angles **q** through the affine relationship 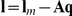 which for constant moment arms also implies 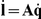. The constant 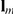 is the reference muscle length when the joint angle is at its rest position (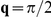). The muscle tension follows a spring-damper model
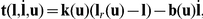
where **k(u)**, **b(u)**, and 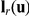 denote the muscle stiffness, the muscle viscosity and the muscle rest length, respectively. Each of these terms depends linearly on the muscle signal **u**, as given by
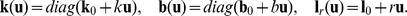
The elasticity coefficient k, the viscosity coefficient b, and the constant r are given from the muscle model of Katayama and Kawato [49]. The same holds true for 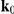, 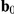 and 
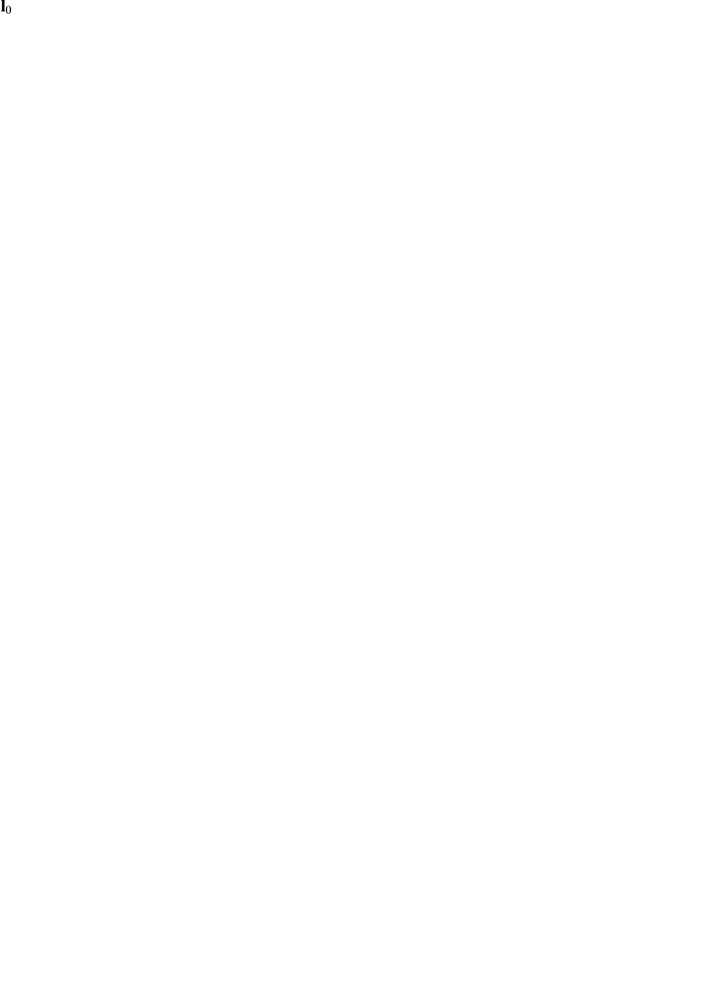
, which are the intrinsic elasticity, viscosity and rest length for 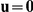, respectively. For exact values please refer to the *Supplementary Information S1.*

To simulate the stochastic nature of neuromuscular signals, often models [33] simply contaminate the neural inputs **u** with multiplicative noise, scaling the kinematic variability proportional to **u**. Such signal-dependent noise cannot account for the complex interplay of neuromuscular noise, modified joint impedance and kinematic variability.

We introduce stochastic information at the level of the muscle tensions by extending the muscle tension function to be
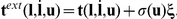
The noise formulation on a muscle level (rather than on a limb level) has the advantage that it can be extended to arm models that incorporate multiple muscles pairs per actuated joint. The variability in muscle tensions depending on antagonistic muscle activations (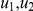) can in a basic form be modeled as an *extended* SDN function:
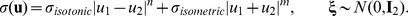
The first term (of the distribution's standard deviation) weighted with a scalar 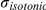 accounts for increasing variability in isotonic muscle contraction (i.e., contraction which induces joint angle motion), while the second term accounts for the amount of variability for co-contracted muscles. The parameters 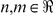 define the monotonic increase of the SDN, which in the literature has been reported to range from less than linear (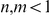), linear (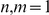) or more than linear (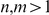). We set 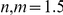 and further make the reasonable assumption that isotonic contraction causes larger variability than pure isometric contraction (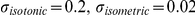). Please note the different absolute value ranges for the isotonic term 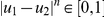 and the isometric term 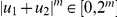 respectively. In reality, at very high levels of co-contraction synchronization effects may occur, which become visible as tremor of the arm [11]. We ignore such extreme conditions in our model. The contraction variability relationship produces plausible muscle tension characteristics without introducing highly complex parameters into the arm model.

To calculate the kinematic variability, the stochastic muscle tensions can be translated into joint accelerations by formulating the forward dynamics including the variability as
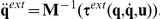
Using the muscle model,
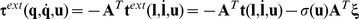
we get an equation of motion including a noise term
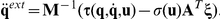
Multiplying all terms leads to following extended forward dynamics equation
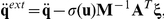
which is separated into a deterministic component 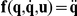 and a stochastic part 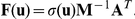
As just shown, the extended SDN corresponds to an additional stochastic term in the joint accelerations which is directly linked to kinematic variability through integration over time. Please note that we introduced this simple but realistic noise model as a surrogate for a more elaborate arm muscle model, which is expected to exhibit such realistic noise-impedance properties [35] as plant behaviour.

One should also note that the stochastic component in our case is only dependent on the muscle signals **u**, because the matrices **A** and **M** are independent of the arm states. However, this can be easily extended for more complex arm models with multiple links or state-dependent moment arms, and our learning algorithm features fully heteroscedastic variances (that is, a possibly state- and control-dependent noise model).

### Finding the optimal control law

Based on the stochastic arm model, let 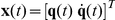 denote the state of the arm model and **u**(t) the applied control signal at time t. We can express the forward dynamics in the presence of noise as
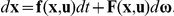
Here, 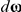 is assumed to be Brownian motion noise, which is transformed by a possibly state- and control-dependent matrix **F**(**x**,**u**). The finite horizon optimal control problem can be stated as follows: Given the initial state 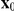 at time t = 0, we seek a (minimal energy) control sequence **u**(t) such that the system's state is at the target 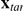 at end-time 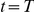. The expected cost, given by the performance index v for such a reaching task (discretized into 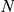 steps, 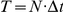 seconds) is of the form
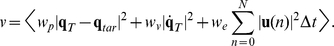
The first term penalizes reaches away from the target joint angle 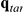, the second term forces a zero velocity at the end time T, and the third term penalizes large muscle commands (i.e., minimizes energy consumption) during reaching. The factors 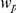, 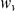, and 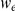 weight the importance of each component. Typical values for a 0.5 seconds simulation are 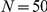 steps with a simulation rate of 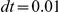.

In order to find the optimal control law we employ an approximate OFC method because the arm dynamics **f** is highly non-linear in **x** and **u** and it does not fit into the Linear Quadratic framework [50]. The iterative Linear Quadratic Gaussian (ILQG) framework [51] is one of the computationally most efficient approximate OFC methods currently available and it supports stochastic dynamics and control boundaries. ILQG iteratively approximates the nonlinear dynamics and the cost function around the nominal trajectory, and solves a locally valid LQG problem to iteratively improve the trajectory. Along with the optimal open loop parameters 
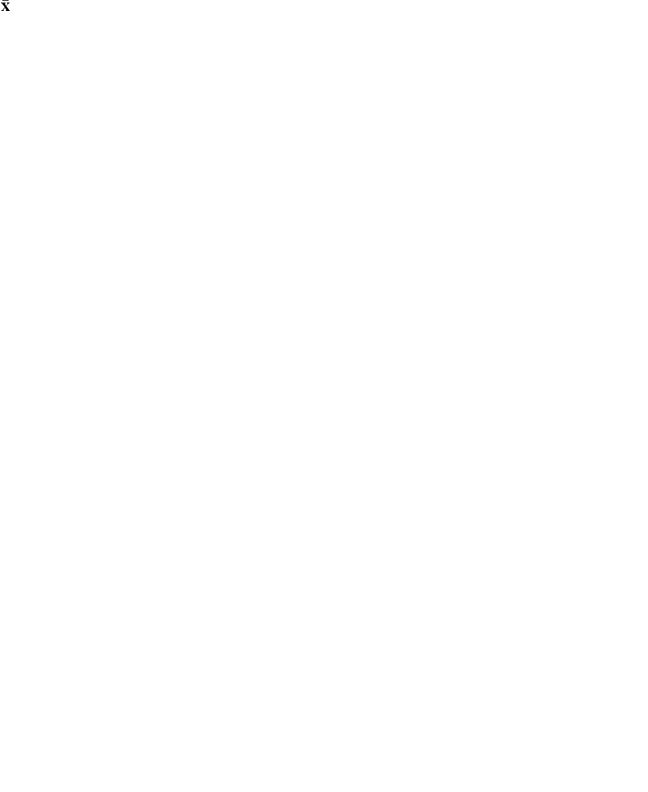
 and 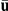, ILQG produces a feedback matrix **L** which serves as locally valid optimal feedback law for correcting deviations from the optimal trajectory on the plant.

It is important to note that the noise model **F(x,u)**, although not visible in the aforementioned cost function v, has an important influence on the final solution because ILQG minimizes the *expected* cost and thereby takes perturbations into account. For a typical reaching-task cost function as described above, this effectively yields an additional (implicit) penalty term that propagates the final cost backwards “through” the uncertainty model. In our case, if at any time the energy cost of activating both muscles is smaller than the expected benefit of being more stable (minimizing uncertainty), then ILQG will command co-contraction. This also explains why our model co-contracts stronger at the final stages of the movement, where noise has a rather immediate impact on the end point accuracy.

### A learned internal model for uncertainty and adaptation

Assuming the internal dynamics model is acquired from sensorimotor feedback then we need to learn an approximation 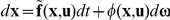 of the stochastic plant forward dynamics 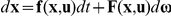. Such problems require supervised learning methods that are capable of **(i)** efficient non-linear regression in an online fashion (important for adaptation) and **(ii)** provide heteroscedastic (i.e., localized) prediction variances in order to represent the stochasticity in the dynamics. As the source of stochasticity, we refer to the kinematic variability of the system described above, which encodes for the uncertainty in the dynamics: if a certain muscle action induces large kinematic variability over trials this will reduce the certainty in those regions. Conversely regions in the state-action space that have little variation will be more trustworthy.

We use *Locally Weighted Projection Regression (LWPR)*, which is a non-parametric incremental local learning algorithm that is known to perform very well even on high-dimensional motion data [52]. Within this local learning paradigm we get access to the uncertainty in form of heteroscedastic prediction variances (*Supplementary information S2*). Once the learning system has been pre-trained thoroughly with data from all relevant regions and within the joint limits and muscle activation range of the arm, a stochastic OFC with learned dynamics (OFC-LD) problem can be formulated that “guides” the optimal solution towards a maximum prediction certainty, while still minimizing the energy consumption and end point reaching error.

The LWPR learner not only provides us with stochastic information originating from internal SDN, but also delivers an uncertainty measure in cases where the dynamics of the arm changes. Notably the internal dynamics model is continuously being updated during reaching with actual data from the arm, allowing the model to account for systematic perturbations [26], for example due to external force fields (FF) (*Supplementary information S3*). This is an extension to previously proposed classic optimal control models that relied on perfect knowledge of the system dynamics, given in closed analytic form based on the equations of motion.

From a computational perspective, the approximative OFC methods currently seem to be the most suitable algorithms available to find OFC laws for nonlinear and potentially high dimensional systems. A limiting factor in OFC-LD is the dynamics learning using local methods, which on the one hand is an important precondition for the availability of heteroscedastic variances but on the other hand suffers from the curse of dimensionality, in that the learner has to produce a vast amount of training data to cover the whole state-action space.

### Simulations

Prior to the reaching experiments, we learnt an accurate forward dynamics model 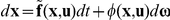 with movement data from our simulated arm *(Supplementary information S4)*. Stochastic ILQG with learned dynamics (ILQG-LD) was used to calculate the optimal control sequence for reaching of duration T = 500 msec with a sampling rate of 10 msec (dt = 0.01). The feedback matrix **L** served as optimal feedback gains of the simulated antagonistic arm.

#### Higher accuracy demands

To model different accuracy demands in OFC, we modulate the final cost parameter 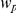 and 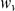 in the cost function, which weights the importance of the positional endpoint accuracy and velocity compared to the energy consumption. We created five different accuracy conditions: (A) 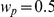, 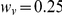; (B) 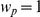, 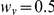; (C) 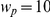, 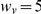; (D) 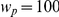, 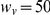; (E) 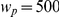, 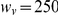; The energy weight for each condition is constant (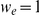). Start position was 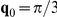 and the target position was 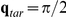. For each condition we performed 20 reaching trials.

#### Higher velocities conditions

Here we set the start position to 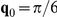 and define three reaching targets with increasing distances: 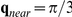; 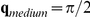; 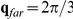. The cost function parameters are 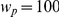, 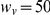, and 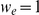. We again performed 20 trials.

#### Adaptation experiments

The reach adaptation experiments were carried out with a constant force acting on the end-effector (i.e., hand). Within all reaching trials, the ILQG-LD parameters were set to: T = 500 msec, 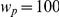, 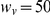, and 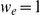, 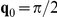, and 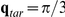. The force-field trials arm dynamics are simulated using a constant force field 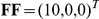 acting in positive x-direction, i.e., in direction of the reaching movement.

## Supporting Information

Supplementary Information S1 (0.08 MB DOC)

Supplementary Information S2 (0.52 MB DOC)

Supplementary Information S3 (0.14 MB DOC)

Supplementary Information S4 (0.04 MB DOC)
