# Supplementary material for: A Computational Model of Limb Impedance Control Based on Principles of Internal Model Uncertainty
Source: PLoS One. 2010 Oct 26;5(10):e13601. doi: 10.1371/journal.pone.0013601 (PMC2964289; doi:10.1371/journal.pone.0013601)
Supplement: Supplementary Information S1 — (0.08 MB DOC) [file pone.0013601.s001.doc]

## Arm and muscle parameters

|  | **Arm parameters** | | |
| --- | --- | --- | --- |
|  |  |  |  |
|  | Link weight [*kg*] | $m=1.59$ | |
|  | Link length [*m*] | $l =0.35$ | |
|  | Center of gravity [*m*] | $L_{g}=0.18$ | |
|  | Moment of Inertia [$kg\cdot m^{2}$ ] | $M=0.0477$ | |
|  | Moment arms [*cm*] | $A=\left[-2.5 2.5\right]^{T}$ | |
|  |  |  |  |
|  | **Muscle parameters** | | |
|  |  |  |  |
|  | Elasticity [*N/m*] | | $k=1621.6$ |
|  | Intrinsic elasticity [*N/m*] | | $k_{o}=810.8$ |
|  | Viscosity [$N\cdot s/m$] | | $b=108.1$ |
|  | Intrinsic viscosity [$N\cdot s/m$] | | $b_{o}=54.1$ |
|  | Rest length constant [*cm*] | | $r=2.182$ |
|  | Intrinsic muscle rest length [*cm*] | | $l_{o}=5.67$ |
|  | Muscle length at rest [*cm*] | | $l_{m}=9.6$ |

Figure S1: Left: Human elbow model with 2 muscles. Right: Used arm and muscle parameters (adapted from [49]). Please note here we defined for convenience of the simulations the rest position as $q_{o}=\pi/2$ whereas in [46] the rest position was defined at $q_{o}=0$. Flexor and extensor muscles are modeled with identical parameters.
