# Supplementary material for: A Computational Model of Limb Impedance Control Based on Principles of Internal Model Uncertainty
Source: PLoS One. 2010 Oct 26;5(10):e13601. doi: 10.1371/journal.pone.0013601 (PMC2964289; doi:10.1371/journal.pone.0013601)
Supplement: Supplementary Information S2 — (0.52 MB DOC) [file pone.0013601.s002.doc]

## The LWPR algorithm and its prediction variances

In LWPR, the regression function is constructed by blending local linear models, each of which is endowed with a locality kernel that defines the area of its validity (also termed its receptive field). During training, the parameters of the local models (locality and fit) are updated using incremental Partial Least Squares, and models can be pruned or added on an as-need basis, for example, when training data is generated in previously unexplored regions. Usually the receptive fields of LWPR are modeled by Gaussian kernels, so their activation or response to a query vector **z** (combined inputs **x** and **u** of the forward dynamics $\tilde{\mathbf{f}}$) is given by

$w_{k}(\mathbf{z})=\exp\left(-\frac{1}{2}(\mathbf{z}-\mathbf{c}_{k})^{T}\mathbf{D}_{k}(\mathbf{z}-\mathbf{c}_{k})\right)$

where $\mathbf{c}_{k}$ is the centre of the *k*-th linear model and $\mathbf{D}_{k}$is its distance metric. Treating each output dimension separately for notational convenience, the regression function can be written as

$$\tilde{\mathbf{f}}(\mathbf{z})=\frac{1}{W}\sum_{k=1}^{K} w_{k}(\mathbf{z})\psi_{k}(\mathbf{z}), W=\sum_{k=1}^{K} w_{k}(\mathbf{z}), \psi_{k}(\mathbf{z})=b_{k}^{0}+\mathbf{b}_{k}^{T}(\mathbf{z}-\mathbf{c}_{k}),$$

where $b_{k}^{0}$and $\mathbf{b}_{k}$ denote the offset and slope of the *k*-th model, respectively.

LWPR learning has the desirable property that it can be carried out online, and moreover, the learned model can be adapted to changes in the dynamics in real-time. A forgetting factor [52], which balances the trade-off between preserving what has been learned and quickly adapting to the non-stationarity, can be tuned to the expected rate of external changes.

The statistical parameters of LWPR regression models provide access to the prediction uncertainties (i.e., variances), here termed *confidence bounds*, of new prediction inputs [52]. In LWPR the predictive variances are assumed to evolve as an additive combination of the variances within a local model and the variances independent of the local model. The predictive variance estimates $\sigma_{pred,k}^{2}$for the *k*-th local model can be computed in analogy with ordinary linear regression. Similarly one can formulate the global variances $\sigma^{2}$ across models. In analogy to the model predictions in $\tilde{\mathbf{f}}(\mathbf{z})$, LWPR then combines both variances additively to form the confidence bounds given by

$$\sigma_{pred}^{2}(\mathbf{z})=\frac{1}{W^{2}}\left(\sum_{k=1}^{K} w_{k}(\mathbf{z})\sigma^{2}+\sum_{k=1}^{K} w_{k}(\mathbf{z})\sigma_{pred,k}^{2}\right).$$

The local nature of LWPR leads to the intuitive requirement that only receptive fields that actively contribute to the prediction (e.g., large linear regions) are involved in the actual confidence bounds calculation. Large confidence bound values typically evolve if the training data contains much noise and other sources of variability such as changing output distributions. Further regions with sparse or no training data, i.e. unexplored regions, show large confidence bounds compared to densely trained regions. Figure S2 depicts the learning concepts of LWPR graphically on a learned model with one input and one output dimension. The noisy training data was drawn from an example function that becomes more linear and noisier for larger ***z***-values. Furthermore in the range ***z*** = [5*..*6] no data was sampled for training to show the effects of sparse data on LWPR learning.


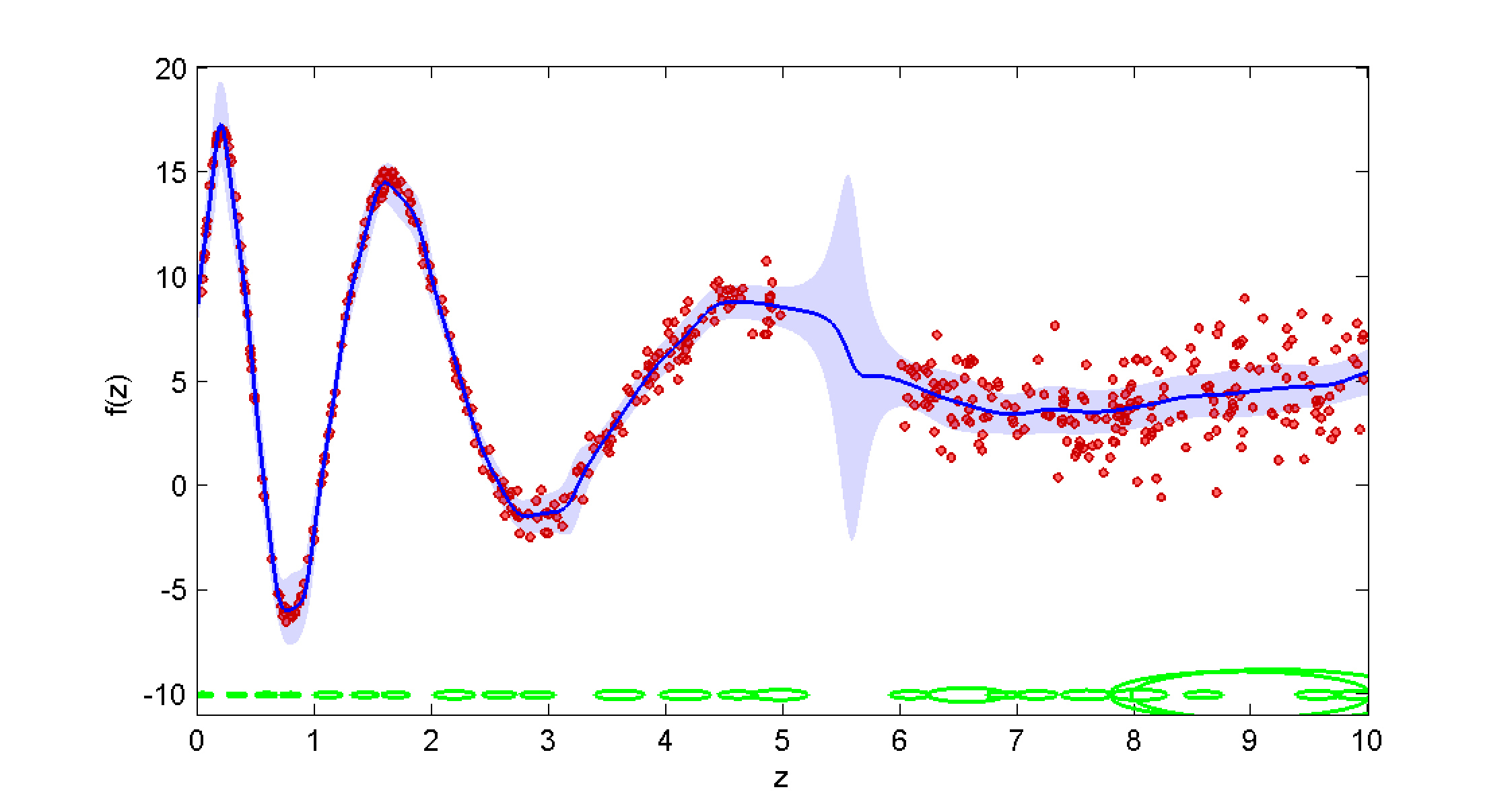


Figure S2: Typical regression function (blue continuous line) using LWPR. The dots indicate a representative training data set. The receptive fields are visualized as ellipses drawn at the bottom of the plot. The shaded region represents the confidence bounds around the prediction function. The confidence bounds grow between z = [5*..*6] (no training data) and generally towards larger z values (noise grows with larger values).
