# Supplementary material for: A Computational Model of Limb Impedance Control Based on Principles of Internal Model Uncertainty
Source: PLoS One. 2010 Oct 26;5(10):e13601. doi: 10.1371/journal.pone.0013601 (PMC2964289; doi:10.1371/journal.pone.0013601)
Supplement: Supplementary Information S3 — (0.14 MB DOC) [file pone.0013601.s003.doc]

## ILQG and ILQG-LD

The ILQG algorithm starts with a time-discretized initial guess of an optimal control sequence and then iteratively improves it w.r.t. the performance criteria

.

From the initial control sequence $\bar{\mathbf{u}}^{i}$ at the *i*-iteration, the corresponding state sequence $\bar{\mathbf{x}}^{i}$is retrieved using the deterministic forward dynamics **f** with a standard Euler integration . In a next step the discretized dynamics are linearly approximated and the cost function $v$ is quadratically approximated around $\bar{\mathbf{x}}_{n}^{i}$*and* $\bar{\mathbf{u}}_{n}^{i}$. The approximations are formulated as deviations of the current optimal trajectory $\delta\mathbf{x}_{n}^{\mathbf{i}}=\mathbf{x}_{n}^{\mathbf{i}}-\bar{\mathbf{x}}_{n}^{\mathbf{i}}$ and $\delta\mathbf{u}_{n}^{i}=\mathbf{u}_{n}^{i}-\bar{\mathbf{u}}_{n}^{i}$ and therefore form a “local” LQG problem. This (stochastic) linear quadratic problem can be solved computationally efficient via a modified Ricatti-like set of equations. The optimization supports constraints for the control variable **u**, such its lower and upper bounds. After the optimal control signal correction $\delta\mathbf{u}^{i}$has been obtained, it can be used to improve the current optimal control sequence for the next iteration using . At last $\bar{\mathbf{u}}_{n}^{i+1}$is applied to the system dynamics and the new total cost along the along the trajectory is computed. The algorithm stops once the cost $v$ cannot be significantly decreased anymore. After convergence, ILQG returns an optimal control sequence $\bar{\mathbf{u}}$ and a corresponding optimal state sequence $\bar{\mathbf{x}}$ (i.e., trajectory). Along with the optimal open loop parameters $\bar{\mathbf{x}}$ and $\bar{\mathbf{u}}$, ILQG produces a feedback matrix **L** which may serve as optimal feedback gains for correcting local deviations from the optimal trajectory on the plant. Implementation details about ILQG can be found in [51].

ILQG-LD extends the algorithm by replacing the analytic internal forward dynamics model by a *learned* model of the system dynamics. The commanded motor signals **u** and the arm positions (e.g., from proprioception) are constantly fed back to the internal model. Like this adaptation scenarios can be modeled and we do not rely anymore on analytic dynamics formulations.


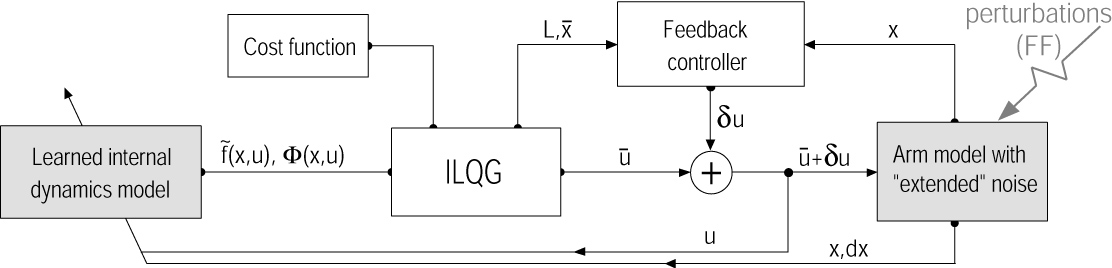


Figure S3: Schematic of the OFC-LD framework using ILQG-LD.

## Details on learning of the internal dynamics model

We coarsely pre-trained an LWPR dynamics model with a data set *S* collected from the arm model *without* using the extended noise model. The data was densely and randomly sampled from the arm's operation range with *,*, and $\mathbf{u}=\left[0,1\right]$. The collected data set ($2.5\cdot10^{6}$data points) was split into a 70% training set and a 30% test set. We stopped learning once the model prediction of $\tilde{\mathbf{f}}\mathbf{ (x},\mathbf{u)}$ could accurately replace the analytic model $\mathbf{f (x},\mathbf{ u)}$, which was checked using *the normalized mean squared error (nMSE)* of $5\cdot10^{-4}$on the test data. After having acquired the noise free dynamics accurately we collected a second data set $S^{noise}$in analogy to *S* but this time the data was drawn from the arm model *including* the extended noise model. We then used $S^{noise}$ to continue learning on our existing dynamics model $\tilde{\mathbf{f}}\mathbf{ (x; u)}$. The second learning round has primarily the effect of shaping the confidence bounds according to the noise in the data and the learning is stopped once the confidence bounds stop changing. One correctly could argue that such a two step learning approach is biologically not feasible because a human learning system for example never gets noise-free data. The justification of our approach is of a practical nature and simplifies the rather involved initial parameter tuning of LWPR and allows us to monitor the global learning success (via the nMSE) more reliably over the large data space. Fundamentally though, our learning method does not conflict with any stochastic OFC-LD principles that we propose.
