# Supplementary material for: A Computational Model of Limb Impedance Control Based on Principles of Internal Model Uncertainty
Source: PLoS One. 2010 Oct 26;5(10):e13601. doi: 10.1371/journal.pone.0013601 (PMC2964289; doi:10.1371/journal.pone.0013601)
Supplement: Supplementary Information S4 — (0.04 MB DOC) [file pone.0013601.s004.doc]

## Details on learning of the internal dynamics model

We coarsely pre-trained an LWPR dynamics model with a data set *S* collected from the arm model *without* using the extended noise model. The data was densely and randomly sampled from the arm's operation range with *,*, and $\mathbf{u}=\left[0,1\right]$. The collected data set ($2.5\cdot10^{6}$data points) was split into a 70% training set and a 30% test set. We stopped learning once the model prediction of $\tilde{\mathbf{f}}\mathbf{ (x},\mathbf{u)}$ could accurately replace the analytic model $\mathbf{f (x},\mathbf{ u)}$, which was checked using *the normalized mean squared error (nMSE)* of $5\cdot10^{-4}$on the test data. After having acquired the noise free dynamics accurately we collected a second data set $S^{noise}$in analogy to *S* but this time the data was drawn from the arm model *including* the extended noise model. We then used $S^{noise}$ to continue learning on our existing dynamics model $\tilde{\mathbf{f}}\mathbf{ (x; u)}$. The second learning round has primarily the effect of shaping the confidence bounds according to the noise in the data and the learning is stopped once the confidence bounds stop changing. One correctly could argue that such a two step learning approach is biologically not feasible because a human learning system for example never gets noise-free data. The justification of our approach is of a practical nature and simplifies the rather involved initial parameter tuning of LWPR and allows us to monitor the global learning success (via the nMSE) more reliably over the large data space. Fundamentally though, our learning method does not conflict with any stochastic OFC-LD principles that we propose.
